# Antidiabetic potential of polysaccharides from *Brasenia schreberi* regulating insulin signaling pathway and gut microbiota in type 2 diabetic mice

**DOI:** 10.1016/j.crfs.2022.09.001

**Published:** 2022-09-07

**Authors:** Gaodan Liu, Simin Feng, Jiadan Yan, Di Luan, Peilong Sun, Ping Shao

**Affiliations:** a Department of Food Science and Engineering, Zhejiang University of Technology, Hangzhou, 310014, Zhejiang, People's Republic of China; b Key Laboratory of Food Macromolecular Resources Processing Technology Research (Zhejiang University of Technology), China National Light Industry, People's Republic of China

**Keywords:** Type 2 diabetes, Microbiota analysis, *Brasenia schreberi*, Polysaccharides, PI3K/Akt signaling pathway

## Abstract

This study aimed to investigate the hypoglycemic activities and gut microbial regulation effects of polysaccharides from *Brasenia schreberi* (BS) in diabetic mice induced by high-fat diet and streptozotocin. Our data indicated that BS polysaccharides not only improved the symptoms of hyperglycemia and relieved metabolic endotoxemia-related inflammation but also optimized the gut microbiota composition of diabetic mice with significantly decreased *Firmicutes/Bacteroidetes* ratios. More importantly, altered gut microbiota components may affect liver glycogen and muscle glycogen by increasing the mRNA expression of phosphatidylinositol-3-kinase (PI3K) and protein kinase B (Akt) in the liver of mice through modulated the abundance of beneficial bacteria (*Lactobacillus*). Altogether, our findings, for the first time, demonstrate that BS polysaccharides may be used as a beneficial probiotic agent that reverses gut microbiota dysbiosis and the hypoglycemic mechanisms of BS polysaccharides may be related to enhancing the abundance of *Lactobacillus* to activate PI3K/Akt-mediated signaling pathways in T2DM mice.

## Highlights

• *Brasenia schreberi* polysaccharides ameliorated hyperglycemia and dyslipidemia in mice.
• The polysaccharides regulated glucose metabolism through activating PI3K-Akt pathway.
• The polysaccharides modulated gut microbiota profile of diabetic mice.

## Graphical abstract

**Figure:**
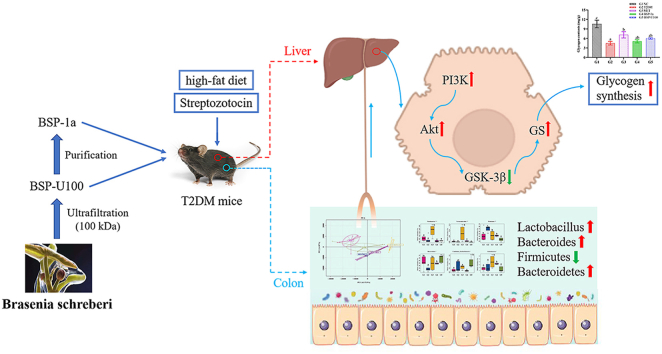

## 1 Introduction

Diabetes mellitus is one of the most common metabolic disorder in the world (Guo et al., 2019), with rising global prevalence over the past 30–40 years (Karlsson et al., 2013). It was reported that about 90% of diabetic patients are clinically diagnosed to be type 2 diabetes mellitus (T2DM) (Liu et al., 2018). Nowadays, the main medicines currently used to treat T2DM are biguanides, thiazolidinediones and sulfonylureas, but many of the synthetic drugs elicit adverse side effects (Chen et al., 2017). Recently, there has been growing interest in the natural anti-diabetic drugs developed from plants (Sun et al., 2020). It is a benefit for patients to combine the different classes of natural substances, which can reduce the risk of developing T2DM, and reduce the risks of adverse side effects from synthetic drugs (Chen et al., 2019).

The major pathogenic factors underlying diabetes are insulin (INS) resistance and *β*-cell dysfunction, which result in high blood glucose levels (Yore et al., 2014). The liver is the main site of INS resistance and INS resistance can lead to elevation of gluconeogenesis and reduction of glycogen synthesis in the liver, and then cause hyperglycemia. Therefore, improving INS resistance is the key to improve the liver injury of T2DM (Liang et al., 2018; Liu et al., 2015). Recently, the associations between common chronic diseases and alteration of intestinal microbial composition and function have attracted a great deal of attention (Huang et al., 2020; Shanmugam et al., 2022). Several factors such as naturally active compounds or drugs can influence the diversity of microbial ecosystems by causing changes in the composition or local distribution of bacterial communities, thereby improving T2DM. (Jovel et al., 2016; Marques et al., 2016; Yang et al., 2022). Recent studies have shown that the gastrointestinal tract is an important target for metformin (MET), and the intestinal microbiota of type 2 diabetes patients improves with MET treatment (Da and Olefsky, 2016). However, MET use in patients with kidney disease remains limited by the perceived, risk of lactic acidosis (Alvarez et al., 2020). Therefore, active compounds derived from natural products, such as polysaccharides, induce structural changes in gut microbiota to improve diabetic symptoms, which has become a potential new treatment for diabetes (Tang et al., 2019). Previous studies have shown that polysaccharides isolated from *Laminaria japonica* can attenuate gestational diabetes by regulating the gut microbiota in mice (Lin et al., 2021).

*Brasenia schreberi* (BS) is an aquatic plant belonging to the Nymphaeaceae family, and is found widely in Asia, North and Central America and Australia. The young leaves of BS are coated with a gelatinous water-insoluble mucilage (Kim et al., 2015).The gelatinous mucilage has large amounts of polysaccharides (Zha et al., 2012). In Asia, BS is considered as a vegetable with many medicinal and nutritive values, and is cultivated and traded. BS was later found exhibiting some biological functions, such as antioxidation (Feng et al., 2019a; Xiao et al., 2016), anti-inflammatory (Legault et al., 2011) and cholesterol-lowering effect (Kim et al., 2015). It was also employed as hypolipidemic supplement for health purposes in food and medicinal industries (Kim et al., 2015). Our previous work revealed a novel α-amylase and α-glucosidase inhibitor derived from BS. This proved that BS polysaccharides can be used as a potential anti-diabetic supplement (Feng et al., 2019b). However, there are few studies on the therapeutic effects of BS on diabetes and its mechanism remain unclear, which greatly hinders its application.

In this study, the aim of this work was to reveal the hypoglycemic mechanism of BS polysaccharides. The fasting blood glucose and INS levels were investigated. Then, the expression of hepatic genes related to glucose metabolism was analyzed. At last, the effects of BS polysaccharides on modulation of intestinal microflora in T2DM mice were also investigated. The hypoglycemic mechanism of BS polysaccharides which related to modulation of intestinal microflora and activation of phosphatidylinositol-3-kinase (PI3K)/protein kinase B (Akt)-mediated signaling pathways was illustrated.

## 2 Materials and methods

### 2.1 Materials

Fresh BS was provided by Hangzhou Qiansheng food factory (Hangzhou, China) and the gel coating surrounding the leaves was stripped off by hand. MET was purchased from Zhonglian Pharmaceutical Co., Ltd. (Shenzhen, China). Streptozotocin (STZ) was purchased from Sigma-Aldrich (St. Louis, MO). The commercial assay kits to measure total cholesterol (TC), total triglyceride (TG), high-density lipoprotein cholesterol (HDL-C), low-density lipoprotein cholesterol (LDL-C), hepatic glycogen, myoglycogen, INS were obtained from Nanjing jiancheng Bioengineering Institute (Nanjing, China). ELISA detection kit for INS was provided by Merck-Millipore (Shanghai, China). Other chemicals used in this study were of analytical grade.

### 2.2 Extraction of polysaccharide from BS

The polysaccharide was isolated and characterized as described in detail in our previous research (Feng et al., 2019b). About 2.5 g BS mucilage was extracted with 3000 mL 0.1 mol/L NaOH at 70 °C for 3 h. After filtered, the extraction was collected. About 1000 mL BSP-NaOH was separated by ultrafiltration membrane (PES10-1812) with 100 kDa, under pressure of 0.5 MPa and the velocity of 30 L/h. The obtained polysaccharide sample was named BSP-U100 polysaccharide (Mw 50–100 kDa) (Fig. S1A). Then the BSP-U100 polysaccharide was concentrated to 10 mg/mL with rotary vacuum evaporator (RE-2000A) and was freeze-dried and stored at −18 °C. The chemical composition of BSP-U100 polysaccharide was shown in Table S1. Total sugar and polyphenols content of BSP-U100 polysaccharide were estimated 79.89% and 18.81%, respectively.

Portion of BSP-U100 polysaccharide was dissolved in distilled water, filtered through 0.45 μm membrane, and then fractioned by a DEAE Sepharose Fast Flow column with distilled water used as eluents to obtain BSP-0, BSP-1, BSP-2 and BSP-3. Compared to other components, BSP-1 component had the highest content. Therefore, the BSP-1 component was selected for subsequent purification using Sephacryl S-500 High Resolution Sephadex S 500 HR (XK 2., 6 mm × 100 mm). As showed in Fig. S1B., the main peak BSP-1a was collected and freeze-dried.

### 2.3 Animal model

**Fig. 1:**
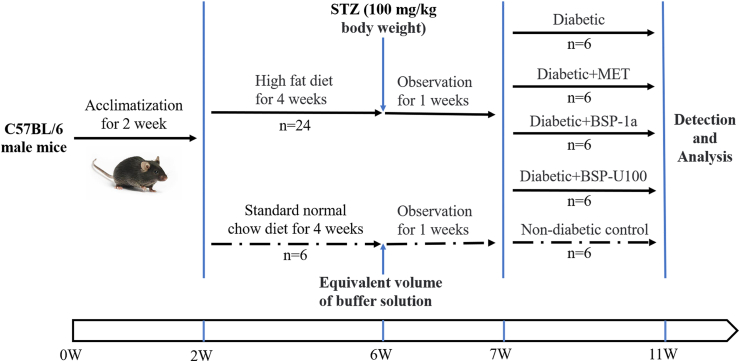
The flow chart of the experiment.

C57BL/6 male mice (4 weeks, weight 20 ± 2 g) of specific pathogen free grade were purchased from Zhejiang Chinese Medical University approved all animal experiments (permit number: Zhe 2019-0010). Mice were housed (6 per cage) in a specific pathogen-free polycarbonate cages in a controlled environment (temperature: 23–25 °C, humidity 70–75%, and a lighting regimen of 12-h light-dark cycles), with free access to food and water. As shown in Fig. 1, after 2 weeks of acclimation, the mice were randomized into two groups: normal control group (NC, n = 6) and an STZ-induced experimental group (n = 24). The NC was fed a normal chow diet (10% of energy as fat, D12450J, Research Diets) throughout the study, whereas the experimental group was fed a high-fat diet (HFD) (60% of energy as fat, D12492, Research Diets) (Coppey et al., 2018; Leguina Ruzzi et al., 2018). After 4 weeks of HFD feeding, experimental mice (24 mice) were fasted overnight (12 h) and induced with diabetes by a single intraperitoneal injection with 3% STZ aqueous solution (dissolved in 0.1 M citrate buffer, pH 4.5) at a dosage of 100 mg/kg (bw), and the NC was injected with the same volume of citrate buffer. One week following the STZ injection, fasting blood glucose (FBG) levels were determined using ACCU-CHEK (R) Active (ROCHE, Germany) and mice with high FBG (>11.1 mmol/L) were considered T2DM mice. Finally, 24 mice were identified to the T2DM model (Gao et al., 2016) and were randomly divided into 4 groups (T2DM, MET, BSP-1a, BSP-U100).

NC group (G1, n = 6) and other groups (T2DM, MET, BSP-1a, BSP-U100) continually fed a normal chow diet. Specifically, mice of G1 and T2DM groups (G2, n = 6) received some volume of distilled water, mice of MET group (G3, n = 6) were administered with 200 mg/kg of body weight of MET once daily; mice of BSP-1a group (G4, n = 6) were administered with 200 mg/kg of body weight of BSP-1a once daily; mice of BSP-U100 group (G5, n = 6) were administered with 200 mg/kg of body weight of BSP-U100 once daily. During the whole experiment, food intake, water intake and body weight were recorded weekly. Every 2 weeks, FBG levels of all mice were monitored using a blood glucose meter (Roche ACCU-CHEK active, Germany).

### 2.4 Biochemical measurements of the serum and hepatic/muscle samples

At the end of the experimental studies, the mice were euthanized by CO_2_ asphyxiation and a blood sample was taken by cardiac puncture. Serum was prepared by centrifuged (3000 rpm, 10 min) and then stored frozen at −80 °C. The serum of TC, TG, HDL-C and LDL-C levels were measured using commercially available assay kits following the manufacturer's instructions (Jiancheng Biological Engineering Institute, Nanjing, China). The liver, colon and muscle were dissected out, weighed and wiped off with ice-cold physiological saline to remove adhering blood. The liver was stored in RNAlater solution (Ambion, Austin, TX, USA) for RNA extraction.

The levels of glucagon, and epinephrine were determined using commercially available assay kits following the manufacturer's instructions (Jiancheng Biological Engineering Institute, Nanjing, China). The levels of INS were determined using ELISA detection kits (Merck-Millipore). The levels of hepatic glycogen and myoglycogen were determined using commercially available assay kits following the manufacturer's instructions (Jiancheng Biological Engineering Institute, Nanjing, China).

### 2.5 Real-time quantitative polymerase chain reaction (RT-qPCR) analysis

Total RNA was isolated from mice’ liver using Trizol Reagent. The concentration of RNA was determined by absorbance at 260 nm. The values of all RNA samples at absorbance 260/280 ranged between 1.8 and 2.0. Total RNA was quantified and reverse-transcribed into cDNA using a Reverse Transcriptase M-MLV (RNase H-) kit (Takara, Japan). The mRNA expressions were then quantified by RT-qPCR using the AceQTM qPCR SYBR Green Master Mix (Takara, Japan) and an Applied Biosystems ViiA 7TM Real-Time PCR System. PCR amplification was performed using the following conditions: initial activation of the hot-start DNA polymerase for 1min at 95 °C followed by 45 cycles (95 °C for 10s and 59 °C for 20 s) (Liang et al., 2018). Gene expression was normalized to the geometric mean of the reference genes (*β*-actin) using the 2^-△△Ct^ method. The specific primers, which include both sense and antisense, were used for the amplification of DNA (Table 1).

**Table 1:** Designed primer sets for RT-PCRa.

gene	primer	sequence (5′-3′)	size (bp)
PI3K	sense	ACAAAGCTCTACTCTAGGCGTG	242
	antisense	TTACCAGCATGGTCATGGGC	
AKT	sense	AGAGAGCCGAGTCCTACAGAATA	133
	antisense	CCGAGAGAGGTGGAAAAACA	
GSK-3β	sense	TCGTCCATCGATGTGTGGTC	202
	antisense	TTGTCCAGGGGTGAGCTTTG	
GS	sense	TTGCCAGAATGCACGCAGAA	270
	antisense	TGCCTGCATCATCTGTTGAC	
β-actin	sense	GATCGATGCCGGTGCTAAGA	367
	antisense	TCCTATGGGAGAACGGCAGA	

### 2.6 Gut microbiota analysis by 16S rDNA high-quality sequencing

After 4 weeks of intervention, randomly select 4 mice in each group to extract genomic DNA from colon contents following the protocol described (Chen et al., 2019) with minor modifications. DNA integrity and quality were evaluated by 1% agarose gel electrophoresis. DNA concentration was measured by NanoDrop (2000) instrument (Thermo Fisher Scientific, USA). The V3–V4 region of the bacterial 16S rRNA gene was amplified using 341F (5′-CCTAGGGNGGCWGCAG-3′) and 805R (5′-GACTACHVGGGTATCTAATCC-3′). The 16S rRNA genes sequencing libraries of bacterial were produced by using a TruSeq DNA PCR-Free Sample Preparation Kit (Illumina, San Diego, CA, USA) for high throughput sequencing. The sequencing was performed on the Illumina MiSeq platform, which was carried out by Genesky Biotechnologies Inc. (Shanghai, China). The paired sequences obtained by paired-end sequencing were spliced using FLASH2 software, and low-quality sequences were removed after the merge sequence was obtained. Mothur software was used to find and remove primers from the sequence. Sequences with a total base error rate greater than 2 and sequences with a length of less than 100 bp were removed using ussearch software to obtain optimized sequences with high quality and reliability (Clean reads), which will be used for subsequent bioinformatics analysis. The finally obtained quality optimization sequences were used for OTU clustering. The alpha diversity of 16S rRNA gene sequences, including observed species, Chao 1, Shannon index, and Simpson index, were calculated with Mothur (v.1.39.3) and R software (v3.4.3). Principal component analysis (PCA) of gut microbiota was performed on R software (v3.4.3). R software was used to analyze the significant differences in microbial community structures between groups at the genus levels.

### 2.7 Statistical analysis

Statistical analysis was performed using SPSS software (SPSS 16.0, Chicago, IL, USA). Data were presented as mean ± standard deviation and analyzed using one-way ANOVA followed by Duncan's new multiple range test among multiple. Differences were considered statistically significant when *P* < 0.05.

## 3 Results

### 3.1 BSP-1a and BSP-U100 polysaccharides ameliorated hyperglycemia and dyslipidemia induced by T2DM

**Fig. 2:**
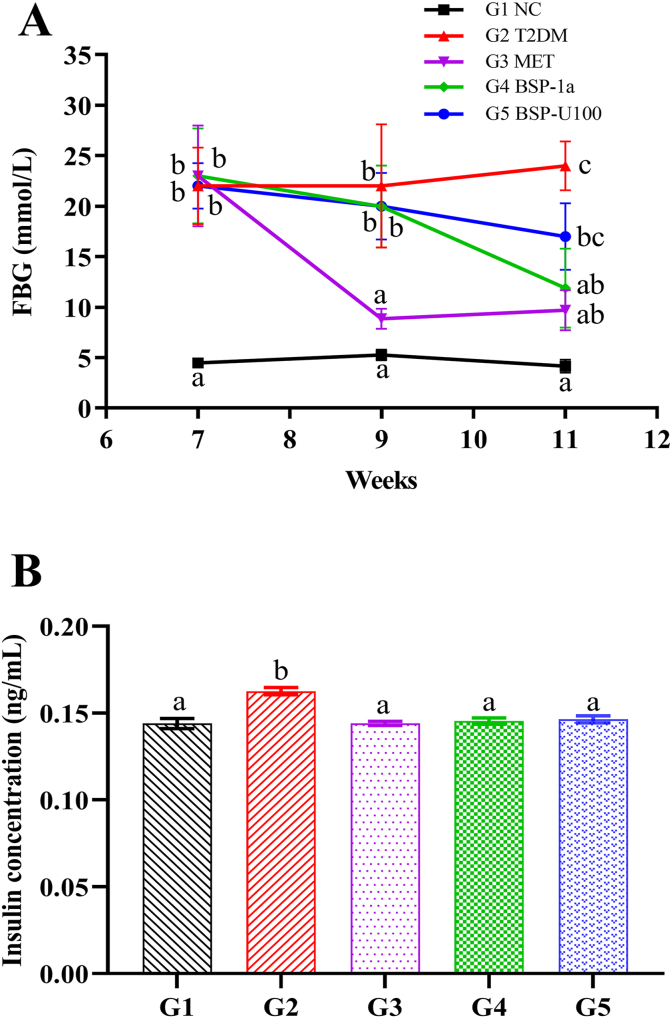
Influence of BSP-1a and BSP-U100 polysaccharides on FBG (A) and insulin (B) in model animals. Significance (*P* < 0.05) among groups is denoted by different letters (n = 6).

We measured the level of FBG during the experiment. As showed in Fig. 2A, at the seventh week, the fasting blood glucose level of diabetic mice was significantly higher than that of NC group (G1) (*P* < 0.01). Moreover, after 11weeks, FBG levels of the BSP-1a group (G4) and BSP-U100 group (G5) were lower than that in the T2DM group (G2), with a reduction rate of 48.30% (*P* < 0.05) and 23.83% (*P* > 0.05), respectively, compared to the initial value.

INS is the primary hormone for regulation of glucose metabolism. INS resistance is typical of T2DM and metabolic syndrome and is often accompanied by hyperinsulinemia and hyperglycemia (Wang et al., 2017; Ye et al., 2019). STZ destroys *β*-cells in pancreatic islets, which causes insufficient INS production and secretion leading to hyperglycemia (Xiao et al., 2017). As showed in Fig. 2B, compared with G1, STZ treatment significantly increased serum INS levels to 0.16 ± 0.01 ng/mL (G2) (*P* < 0.01). It indicated the development of INS resistance. After treatment with MET and BS polysaccharides, serum INS levels were reduced (0.14 ± 0.01 ng/mL by MET, 0.15 ± 0.01 ng/mL by BSP-U100 polysaccharide, 0.15 ± 0.01 ng/mL by BSP-1apolysaccharide) (*P* < 0.01).

In Table 2, the results show that there is no significant difference in the mouse body weight among groups (*P* > 0.05). After 4 weeks of intervention, compared with G2, the water consumption of MET group (G3) decreased by 72.52% and dramatically quenched the thirst for water (*P* < 0.01). Noticeably, the water consumption of mice treated with BSP-1a and BSP-U100 polysaccharides was not significantly different from that of T2DM mice (*P* > 0.05). Compared with the G1, TC (7.92 mmol/L) and LDL-C (0.37 mmol/L) levels of T2DM mice were elevated (*P* < 0.05). Under MET or BS polysaccharides treatments, TC and LDL-C in T2DM mice returned to normal levels. There were no observed differences in TG and HDL-C content associated with the BS polysaccharide treatment.

**Table 2:** Effects of BSP-1a and BSP-U100 polysaccharide on biochemical indicators of mice for 11 weeks.

Parameter	Body weight (g)	Water intake (g)	TC (mmol/L)	TG (mmol/L)	HDL-C (mmol/L)	LDL-C (mmol/L)
G1	25.95 ± 1.52^a^	25.03 ± 1.82^a^	4.42 ± 0.60^a^	0.50 ± 0.17^a^	1.80 ± 0.44^a^	0.13 ± 0.01^a^
G2	21.58 ± 2.33^a^	112.68 ± 4.01^b^	7.92 ± 0.48^b^	0.61 ± 0.12^a^	1.93 ± 0.07^a^	0.37 ± 0.06^b^
G3	23.08 ± 0.74^a^	30.96 ± 1.73^a^	4.37 ± 1.09^a^	0.52 ± 0.13^a^	2.21 ± 0.27^a^	0.14 ± 0.13^a^
G4	23.08 ± 1.53^a^	110.03 ± 3.51^b^	3.71 ± 0.21^a^	0.39 ± 0.11^a^	1.86 ± 0.20^a^	0.13 ± 0.02^a^
G5	22.32 ± 2.84^a^	111.22 ± 4.24^b^	4.85 ± 1.07^a^	0.40 ± 0.13^a^	1.84 ± 0.42^a^	0.11 ± 0.02^a^

Values are presented as means ± SD for n = 5. Values sharing a common letter (a, b, c, d and e) in each comparison did not show any statistically significant differences accessed by one-way ANOVA (p < 0.05).

G1, NC; G2, T2DM; G3, MET; G4, BSP-1a; G5, BSP-U100.

TC, total cholesterol; TG, total triglyceride; HDL-C, high-density lipoprotein; LDL-C, low-density lipoprotein.

### 3.2 BSP-1a and BSP-U100 polysaccharides promoted the synthesis of glycogen in diabetic mice

**Fig. 3:**
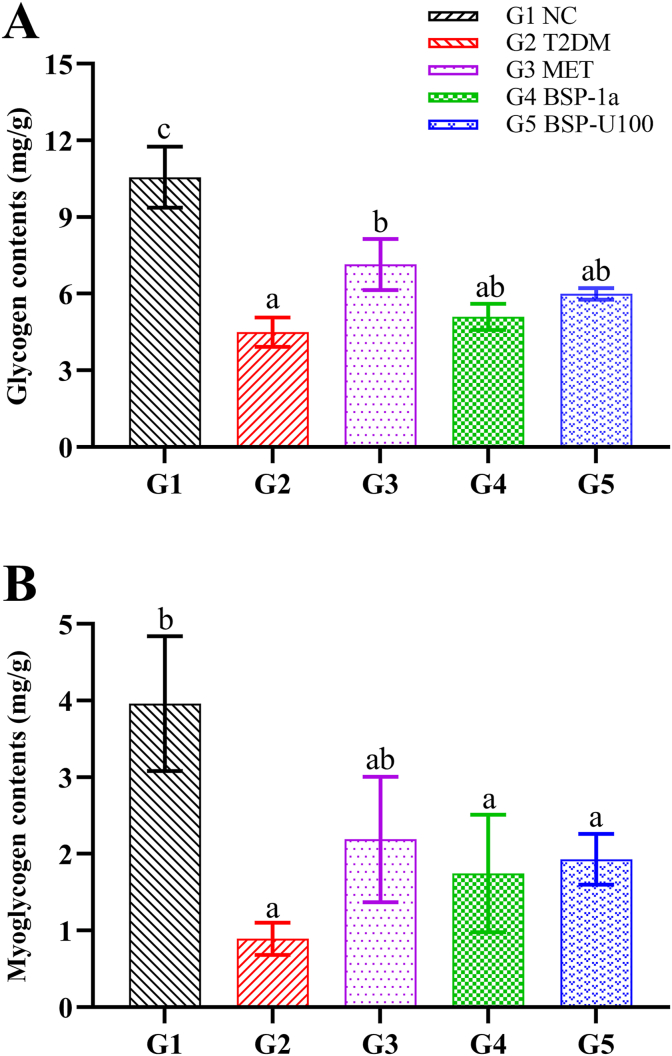
Effect of BSP-1a and BSP-U100 polysaccharides on glycogen (A) and myoglycogen (B) in diabetic mice. Significance (*P* < 0.05) among groups is denoted by different letters (n = 6).

In order to further understand the hypoglycemic effect caused by the increase of liver glycogen and muscle glycogen, the contents of liver glycogen and muscle glycogen in all groups were measured at the 11th week. As illustrated in Fig. 3A, compared with NC group (G1), the glycogen level of T2DM mice (G2) was significantly reduced, from 10.56 ± 1.19 mg/g to 4.49 ± 0.58 mg/g (*P* < 0.01). After 4 weeks of treatment with MET, the level of liver glycogen increased to 7.15 ± 1.00 mg/g (*P* < 0.05). The treatment of BSP-1a and BSP-U100 polysaccharides can increase the level of glycogen in T2DM mice by 13.36% and 33.41%, respectively. The treatment of MET and BS polysaccharides can increase muscle glycogen levels in T2DM mice (Fig. 3B). It was worth noting that the effect of BS polysaccharides in improving liver and muscle glycogen levels was inferior to MET treatment, and the therapeutic effects of BSP-1a and BSP-U100 polysaccharides were not significantly different.

### 3.3 BSP-1a and BSP-U100 polysaccharides affected the PI3K/Akt signal pathway in the liver tissue of diabetic mice

**Fig. 4:**
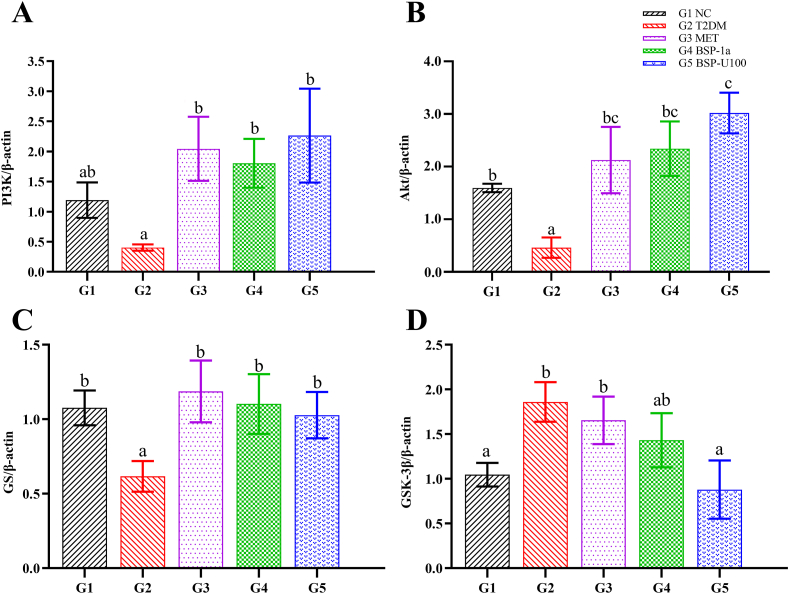
Effect of BSP-1a and BSP-U100 polysaccharides on the expression of PI3K/Akt pathway in diabetic mice. Significance (*P* < 0.05) among groups is denoted by different letters (n = 6).

To investigate the mechanism of BSP-1a and BSP-U100 polysaccharides ameliorating the symptoms of T2DM, mRNA levels of PI3K/Akt signaling pathway in diabetic mice were evaluated by RT-PCR. As showed in Fig. 4, the expression level of PI3K, Akt and glycogen synthase (GS) in liver tissue of T2DM group (G2) was decreased by 66.01% (*P* < 0.05, by ANOVA), 71.02% (*P* < 0.05, by ANOVA), 42.69% (*P* < 0.05, by ANOVA) compared with G1, respectively. Compared with G1, the expression level of glycogen synthase kinase-3β (GSK-3*β*) in liver tissue of G2 was significantly higher than that in G1 by 77.87% (*P* < 0.05, by ANOVA). After supplied with polysaccharides for four weeks, the expression level of PI3K, Akt and GS were obviously increased (*P* < 0.05). The results showed that BSP-1a polysaccharides increased the expression of PI3K, Akt and GS to 4.46-fold, 5.07-fold and 1.79-fold of G2, respectively, while GSK-3β decreased to 0.77-fold of G2. Meanwhile, BSP-U100 polysaccharides increased the expression of PI3K, Akt and GS to 5.56-fold, 6.53-fold,1.67-fold of G2, respectively, while GSK-3β decreased to 0.47-fold of G2. Noticeably, BSP-1a polysaccharides was more effective than BSP-U100 polysaccharides on the downregulation of GSK-3*β* in T2DM mice (*P* < 0.01).

### 3.4 BSP-1a and BSP-U100 polysaccharides administration modulated gut microbiota

**Fig. 5:**
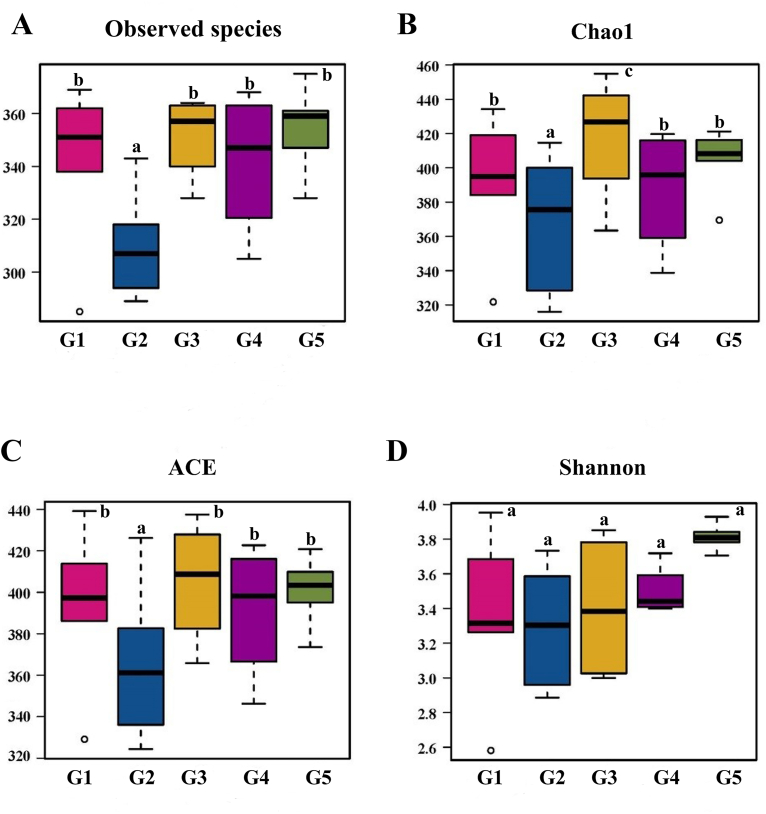
Microbial diversity and difference in the bacterial community between groups categorized according to BSP-1a and BSP-U100 polysaccharides. The alpha diversity analysis on de-noised sequences of mice fecal microbiota with observed species respectively (A), Chao1index (B), ACE (C), and Shannon diversity (D). Significance (*P* < 0.05) among groups is denoted by different letters (n = 4).

High-quality Illumina HiSeq sequencing of the V3–V4 region of the 16S rRNA gene was used to investigate the structural changes in the gut microbiota. α-diversity analysis is an important part of microbial diversity analysis. It mainly focuses on the degree of diversity of species in Locally homogeneous environment, and conducts species richness and diversity research on a sample or multiple samples (Koh, 2018). We examined the α-diversity of the intestinal microbiota in mice and found Observed species, Chao1 and ACE indices were significantly lower in G2 than G1 (Fig. 5A–C) (*P* < 0.01). However, the Shannon index showed no significant difference between two groups (Fig. 5D) (*P* > 0.05). This indicated that the community richness of T2DM mice was reduced, but the diversity of the gut microbiota was stable. Meanwhile, compared with G2, the species abundance of G3, G4, and G5 were higher than that of the G1 (*P* < 0.01), indicating that MET and BS polysaccharides had the ability to increase bacterial richness.

**Fig. 6:**
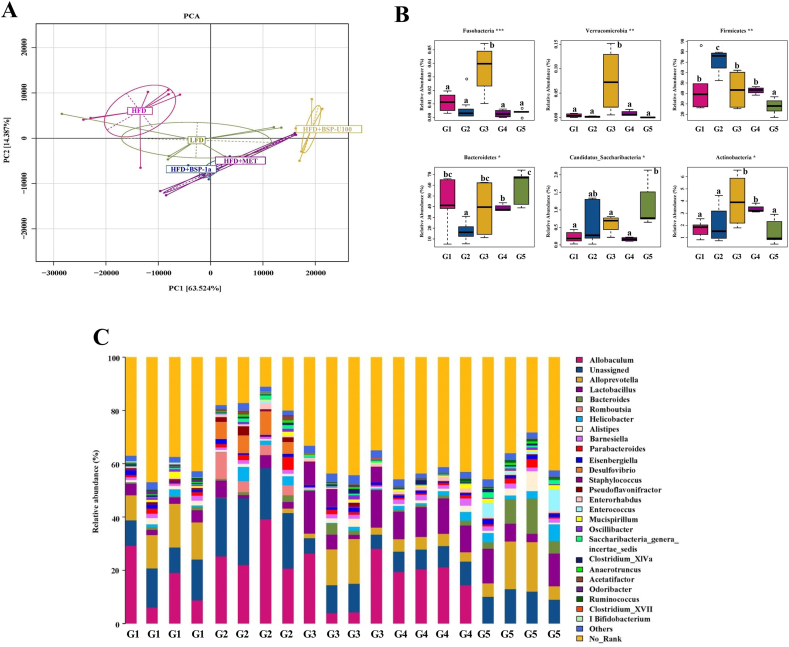
BSP-1a and BSP-U100 polysaccharides reverse gut microbiota dysbiosis in diabetic mice. Principal component analysis (PCA) of gut microbiota in mice (A), microbial community at the phylum level of mice (B) and changes in microbial community at the genus level of mice (C). Significance (*P* < 0.05) among groups is denoted by different letters (n = 4).

PCA was used to analyze changes in the distribution of gut microbiota composition by BSP-1a and BSP-U100 polysaccharides. PCA score plot showed that the intestinal microbiota from the G1, G2, G3, G4 and G5 were highly distinct in organismal structure (Fig. 6A). As the modulation effect of MET on gut microbiota has been well documented in previous studies (Wu et al., 2017; Zhang et al., 2015). The PCA scores showed that the gut microbiota in G2 exhibited a significant structural shift along the negative direction of the first principal component (PC1) compared with the G1. However, supplementation with BSP-1a and BSP-U100 polysaccharides markedly reverted the HFD-induced variations along the positive direction of PC1. Hierarchical clustering plot also revealed that the composition of colon microbiota in mice of G3 and G5 was more similar to that of G1. In general, administration of BSP-1a and BSP-U100 polysaccharides were beneficial to improve colon microbiota compositions in T2DM mice.

The bacterial abundance in each group was determined to investigate composition alterations in the gut microbiota. From the level of phylum, two dominant phyla (*Bacteroidetes* and *Firmicutes*) were shared by all mice groups but varied in their relative abundance (Fig. 6B). Compared with G1, lower *Bacteroidetes* and higher *Firmicutes* abundance were observed in G2. In addition, compared with G1, an increased *Firmicutes* to *Bacteroidetes* ratio (F/B ratio) was observed in G2 (Table 3). Besides, the BSP-1a and BSP-U100 polysaccharides treatment significantly reversed the F/B ratio, and the ratio of G4 was close to that in G1 (*P* < 0.01).

**Table 3:** Changes of relative abundance ratio of phylum Firmicutes to Bacteroidetes ratio (F/B ratio).

	G1	G2	G3	G4	G5
F/B ratio	1.05 ± 0.01^b^	4.12 ± 0.01^c^	1.14 ± 0.04^b^	1.11 ± 0.03^b^	0.48 ± 0.04^a^

Values are presented as means ± SD for n = 5. Values sharing a common letter (a, b and c) in each comparison did not show any statistically significant differences accessed by one-way ANOVA (p < 0.05).

G1, NC; G2, T2DM; G3, MET; G4, BSP-1a; G5, BSP-U100.

Taxon-based analysis also revealed specific bacterial phylotypes among the five groups, including the G1, G2, G3, G4 and G5 (Fig. 6C). As compared with the G1, the level of *Allopravotella* and *Lactobacillus* was decreased (*P* < 0.05), while the level of *Allobaculum*, *Romboutsia* and *Desulfovibrio* increased in the G2 (*P* < 0.05). However, both BSP-1a and BSP-U100 polysaccharides obviously enriched the amount of *Allopravotella*, *Lactobacillus* and *Bacteroides* (*P* < 0.01), and decreased the relative abundance of *Romboutsia* and *Desulfovibrio* (*P* < 0.05). In addition, we found that the number of *Allobaculum* decreased significantly in G5 (*P* < 0.01), but did not change significantly in G4 (*P* > 0.05). Our results indicated that BS polysaccharides optimized the gut microbiota composition of diabetic mice effectively by increasing the abundance of beneficial bacteria and decreasing the conditional pathogenic bacteria.

## 4 Discussion

In this study, BSP-1a and BSP-U100 polysaccharides were used in investigating the beneficial effects on T2DM mice. Decreasing glycaemia is an important indicator for successful diabetes treatments. Prolonged exposure of tissues to supraphysiological levels of blood glucose can lead to adverse outcomes (Ceriello, 2012). The administration of BS polysaccharides has been shown to reduce FBG and serum INS levels, indicating that BS polysaccharides had antidiabetic effects. However, the BS polysaccharides did not completely restore the blood glucose concentrations to normal levels. Dyslipidemia is commonly occurred in T2DM patients and a metabolic disorder characterized by abnormal levels of TC, TG, LDL-C and HDL-C (Tangvarasittichai, 2015). Significant decrease in the level of TC and LDL-C in diabetic mice after administration of BSP-U100 and BSP-1a polysaccharide indicated that the disordered lipid metabolism in diabetic mice was improved.

Liver, the most important organ in the regulation of glucose metabolism, assimilates increased blood glucose in the form of glycogen (Yoon et al., 2001). In the present study, the liver glycogen level was higher in the G1 compared with G2 as the high load of blood glucose was mobilized to and accumulated in the liver. And the PI3K/Akt signaling transduction pathway plays a crucial role in regulating glucose metabolism (Krook et al., 2004). GSK-3*β*, which is an important substrate of Akt, is a key regulatory kinase in glucose metabolism. GSK-3*β* inhibits the activity of GS by phosphorylation, decreasing glycogen synthesis and elevating the blood glucose level (Cohen and Frame, 2001). Akt-induced GSK-3*β* phosphorylation may improve liver glycometabolism by promoting glycogen synthesis. In this study, after administration of BSP-1a and BSP-U100 polysaccharide, phosphorylated Akt effectively regulated the process of glycogen synthesis by inhibiting the phosphorylation of GSK-3β. At the same time, hepatic GS was also dephosphorylated to restore the activity of catalyzing hepatic glycogen synthesis. Then, an increase in the hepatic glycogen synthesis causes a decrease in blood sugar. Therefore, BSP-1a and BSP-U100 polysaccharide can promote the PI3K/Akt signaling pathway of insulin. In addition, with BSP-U100 polysaccharide supplements, the mice have higher synthesis of liver glycogen and muscle glycogen. This indicated that BSP-U100 polysaccharide exerts more positive amelioration on T2DM mice than BSP-1a polysaccharide, which may be related to the fact that it contains polyphenols. A lot of evidence shows that polyphenol supplementation can also activate the PI3K/Akt pathway to increase glycogen synthesis (Kim et al., 2019; Li et al., 2020; Xia et al., 2021).

Intestinal flora, the largest micro-ecological system in the human body, multiple evidence have shown that a series of metabolic diseases of human may be related to intestinal microecological disorders including diabetes (Karlsson et al., 2013; Qin et al., 2012). Larsen et al. found that the abundance of phylum *Firmicutes* in the human gut were significantly reduced in patients with diabetes, and the *Firmicutes* to *Bacteroidetes* ratio (F/B ratio) was found negatively correlated to the level of blood glucose (Larsen et al., 2010). Compared with the G1, an increased *Firmicutes* to *Bacteroidetes* ratio was observed in the G2, which was reported to be correlated to the high FBG and inflammation status (Song et al., 2015). Furthermore, the decreased F/B ratio, which was reported to be associated with the improvement of metabolic disorder (Turnbaugh et al., 2006), was observed in the gut microbiota of BSP-1a and BSP-U100 polysaccharides treated group.

And we found that the abundance of *Lactobacilli* also significantly increased after polysaccharide supplementation (Fig. 6C). As a probiotic dietary supplement, researchers found that *Lactobacillus* has anti-diabetic potential. *Lactobacillus casei* administration favorably regulated blood glucose balance, improve glucose tolerance and also improved lipid metabolism. And it can improve INS resistance by upregulating mRNA expression of PI3K and GS and inhibited the gene expression of GSK-3*β* in the liver (Li et al., 2017; Wang et al., 2017). *Lactobacillus paracasei* TD062 could promote the glucose uptake and consumption by the PI3K/Akt pathway and regulate the levels of FBG, glucose tolerance, hepatic glycogen and lipid metabolism (Dang et al., 2018). *Lactobacillus paracasei* 1F-20 and *Lactobacillus fermentum* F40-4 can increase the uptake of glucose by up-regulating the protein expression of PI3K and pAKT (Zhang et al., 2020). Based on previous research, the anti-diabetic effects of BSP-1a and BSP-U100 polysaccharides might be attributed to the up-regulation of *Lactobacillus* in the gut microbiota, which activates the PI3K/AKT signaling pathway.

Interestingly, we found that the abundance of *Lactobacillus* in mice supplemented with BSP-U100 polysaccharide was lower than that of mice supplemented with BSP-1a polysaccharide, and the abundance of *Bacteroides* was significantly higher than that of mice supplemented with BSP-1a polysaccharide. This may be due to BSP-U100 polysaccharides contain polyphenols and other substances. Similar phenomena have also been found in other experiments exploring the effects of polyphenols on the gastrointestinal flora (Ma and Chen, 2020; Wu et al., 2018). And the presence of polyphenols may have also caused the increase in the number of Allobaculum in G4 mice compared with G5 (Jiao et al., 2019; Van Hul et al., 2018).

## 5 Conclusions

In conclusion, the results demonstrated that BSP-1a and BSP-U100 polysaccharides had the potential to decrease blood glucose levels, improve the serological parameters associated with diabetes. This experimental study found that BSP-1a and BSP-U100 polysaccharide can enhance the insulin-PI3K/Akt signaling pathway, thus promoting glucose transport and liver glycogen synthesis. In addition, BSP-1a and BSP-U100 polysaccharides also showed a probiotic effect on the intestinal microflora of type 2 diabetic mice. This study indicated that BSP-1a and BSP-U100 polysaccharide treatment can significantly increase the beneficial bacteria (*Lactobacillus*) in the intestine of diabetic mice, further increase the expression of PI3K and Akt in the liver of diabetic mice and improve glucose metabolism. This study provides a scientific basis for the application of BS polysaccharides for develops novel natural hypoglycemic products.

## CRediT authorship contribution statement

**Gaodan Liu:** Methodology, Formal analysis, Visualization, Writing – original draft. **Simin Feng:** Writing – review & editing, Data curation. **Jiadan Yan:** Writing – original draft, Data curation. **Di Luan:** Methodology. **Peilong Sun:** Investigation. **Ping Shao:** Writing – review & editing.

## Declaration of competing interest

The authors declare that they have no known competing financial interests or personal relationships that could have appeared to influence the work reported in this paper

## Abbreviations used

Akt: protein kinase b
BS: brasenia schreberi
FBG: fasting blood glucose
GS: glycogen synthase
GSK-3*β*: glycogen synthase kinase-3*β*
HDL-C: high-density lipoprotein cholesterol
HFD: high-fat diet;
INS: insulin
LDL-C: low density lipoprotein cholesterol
LFD: low-fat diet;
PCA: principal component analysis
PC1: first principal component
PI3K: phosphatidylinositol 3-kinase
RT-Qpcr: real-time quantitative polymerase chain reaction
STZ: streptozotocin
T2DM: type 2 diabetes mellitus
TC: cholesterol
TG: triglyceride.
