# Supplementary material for: Antidiabetic potential of polysaccharides from Brasenia schreberi regulating insulin signaling pathway and gut microbiota in type 2 diabetic mice
Source: Curr Res Food Sci. 2022 Sep 7;5:1465–74. doi: 10.1016/j.crfs.2022.09.001 (PMC9478496; doi:10.1016/j.crfs.2022.09.001)
Supplement: Multimedia component 1 [file mmc1.docx]

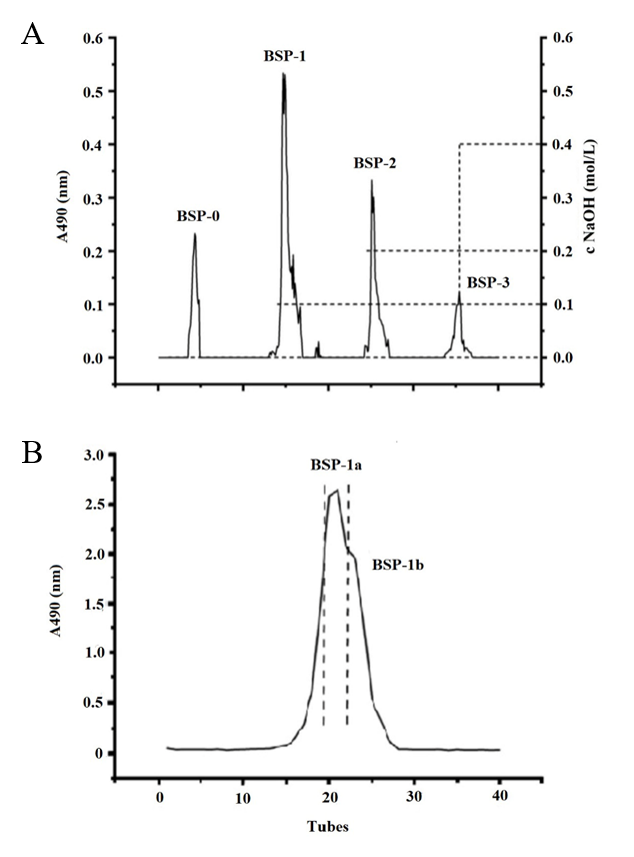


**Fig. S1.** DEAE Sepharrose Fast Flow chromatography of BSP-U100 (A) and chromatography of BSP-1a by Sephacryl S500 (B).

**Table S1.** Physicochemical characterization of BSP-U100 polysaccharides

| total sugar (mg/mL) | reducing sugar(mg/mL) | protein (mg/mL) | flavonoids (mg/mL) | polyphenols (mg/mL) |
| --- | --- | --- | --- | --- |
| 137.75 ± 0.56^c^ | 0.27 ± 1.5^a^ | 1.54 ± 0.03^a^ | 0.43 ± 0.89^a^ | 32.44 ± 0.29^b^ |

Data are expressed as means ± standard deviation (SD; n = 3). Significance (P < 0.05) among groups is denoted by different letters.
